# ISOexpresso: a web-based platform for isoform-level expression analysis in human cancer

**DOI:** 10.1186/s12864-016-2852-6

**Published:** 2016-08-12

**Authors:** In Seok Yang, Hyeonju Son, Sora Kim, Sangwoo Kim

**Affiliations:** 1 Severance Biomedical Science Institute, Yonsei University College of Medicine, 50-1 Yonsei-ro, Seoul, 03722 Korea; 2 Brain Korea 21 PLUS Project for Medical Sciences, Yonsei University College of Medicine, 50-1 Yonsei-ro, Seoul, 03722 Korea

**Keywords:** Differential expression, Isoform, Tissue, Tumor-specific isoform

## Abstract

**Background:**

Alternative splicing events that result in the production of multiple gene isoforms reveals important molecular mechanisms. Gene isoforms are often differentially expressed across organs and tissues, developmental stages, and disease conditions. Specifically, recent studies show that aberrant regulation of alternative splicing frequently occurs in cancer to affect tumor cell transformation and growth. While analysis of isoform expression is important for discovering tumor-specific isoform signatures and interpreting relevant genomic mutations, there is currently no web-based, easy-to-use, and publicly available platform for this purpose.

**Description:**

We developed ISOexpresso to provide information regarding isoform existence and expression, which can be grouped by cancer vs. normal conditions, cancer types, and tissue types. ISOexpresso implements two main functions: First, the Isoform Expression View function creates visualizations for condition-specific RNA/isoform expression patterns upon query of a gene of interest. With this function, users can easily determine the major isoform (the most expressed isoform in a sample) of a gene with respect to the condition and check whether it matches the known canonical isoform. ISOexpresso outputs expression levels of all known transcripts to check alterations of expression landscape and to find potential tumor-specific isoforms. Second, the User Data Annotation function supports annotation of genomic variants to determine the most plausible consequence of a variation (e.g., an amino acid change) among many possible interpretations. As most coding sequence mutations are effective through the subsequent transcription and translation, ISOexpresso automatically prioritizes transcripts that act as backbones for mutation effect prediction by their relative expression. By employing ISOexpresso, we could investigate the consistency between the most expressed and known canonical/principal isoforms, as well as infer candidate tumor-specific isoforms based on their expression levels. In addition, we confirmed that ISOexpresso could easily reproduce previously known isoform expression patterns: recurrent observation of a major isoform across tissues, differential isoform expression patterns in a given tissue, and switching of major isoform during tumorigenesis.

**Conclusions:**

ISOexpresso serves as a web-based, easy-to-use platform for isoform expression and alteration analysis based on large-scale cancer database. We anticipate that ISOexpresso will expedite formulation and confirmation of novel hypotheses by providing isoform-level perspectives on cancer research. The ISOexpresso database is available online at http://wiki.tgilab.org/ISOexpresso/.

**Electronic supplementary material:**

The online version of this article (doi:10.1186/s12864-016-2852-6) contains supplementary material, which is available to authorized users.

## Background

Alternative splicing occurs in most human genes with multiple exons as a regulatory process of gene expression [1]. In this process, particular exons of a gene may be included or excluded in the final processed mRNAs to produce multiple distinct transcript isoforms, bringing great diversity to human proteins [2]. To date, several mechanisms for alternative splicing have been reported, such as alternative promoters, exon skipping, mutually exclusive exons, exon scrambling, alternative 5^′^ and 3^′^ splice sites, retained introns, and alternative polyadenylation [1, 2]. Aberrant splicing patterns that are commonly observed in human cancers have been associated with splicing regulators [2, 3]. For instance, dysregulated expression of splicing regulators (e.g., RBFOX2, PTB/PTBP1, and SRSF1 genes) has been reported to cause splicing pattern changes in genes [3, 4].

The advent of whole transcriptome sequencing (RNA-seq) and development of related bioinformatics analysis tools have enabled us to observe not only the expression snapshot of genes but also their sequences and structural configurations. Particularly, several computational methods have successfully identified the presence of multiple isoforms to reconstruct the composition of mixed transcripts. RSEM [5] and eXpress [6] adopt ungapped alignment of reads to reference transcriptome and estimate transcript abundance by the expectation-maximization (EM) algorithm. Cufflinks [7] uses gapped (spliced) alignment against reference genome as an input to build the overlap graph and to compute the minimum path cover for estimating the presence and abundance of transcripts. StringTie [8] performs read alignment with a procedure similar to that of Cufflinks, builds flow network for path with the heaviest coverage, and then computes maximum flow to estimate the abundance of each transcript. Sailfish [9] employs an alignment-free approach using k-mer indexed transcripts, and determines maximum likelihood estimates of relative transcript abundance by counting the indexed k-mers in the set of raw reads and applying the EM algorithm. When applied to cancer, disease-specific formation of alternative transcripts could be identified as potential biomarkers for diagnosis [4, 10], cancer subtyping [11, 12], and tumor vaccine targets for immunotherapeutics [10].

While the methodological robustness for isoform-level analysis is being gradually improved, the demand for convenient applications to analyze cancer genomes remains, particularly for large-scale databases. First, information regarding isoform presence and expression is hardly centralized and visualized for easy access. Many online expression databases such as TiGER [13], BioGPS [14], BioXpress [15], and Gene Expression Atlas [16] allow users to inspect genome-scale expression profiles at the gene level. GeneFriends [17] and MIsoMine [18] are the only databases that currently utilize transcript-level expression information. However, GeneFriends is developed for the sole aim to identify co-expressed genes (and transcripts), and MIsoMine provides tissue-specific isoform expression information only for mice. A more generalized platform on which differential transcript expression can be aggregated and compared across multiple tumor tissues would serve as a valuable platform for hypothesis formulation and testing. Second, nomenclature for genes, transcript isoforms, and their relationships should be carefully considered and handled, because inappropriate annotations often make simple questions far more complicated. Determining how many isoforms exist in a gene, which genomic regions are protein-coding, and which transcript should be considered as the representative form are typical inherent problems in an isoform-level analysis. Third, transcript expression information can be utilized for interpreting genomic sequence variations. Conventional annotation of genomic mutations usually outputs all possible amino acid changes with respect to the known isoforms. When different isoforms are considered, different consequences can be predicted at the protein level. Once the major isoform is identified, we can resolve this ambiguity for more accurate annotation.

To meet the demands, we developed ISOexpresso, a web-based, graphical, and multi-purpose platform for isoform-level analysis in cancer. ISOexpresso provides isoform-level expression profiles for genes of interest and creates visualizations with a convenient user interface, such as expression statistics and chart display, thereby helping users detect condition-specific isoform expression (e.g., tumor vs. normal). ISOexpresso is built upon standard nomenclature used by many external databases including the University of California Santa Cruz (UCSC) table browser, the Consensus Coding Sequences (CCDS), Ensembl, the Reference Sequence (RefSeq), and the Universal Protein Resource (UniProt), and efficiently harnesses useful information of different sources such as biotype, known canonical isoform, genomic location, and exon structure. ISOexpresso also provides isoform-specific variant annotations prioritized by expression level to predict the most plausible functional consequences of the genomic mutations in the given sample. By using ISOexpresso, we found that a significant number of non-canonical isoforms were expressed in the cancer samples to cause “switching” of major isoforms. In addition, we could easily reproduce previous discoveries on cancer isoform expression as a use-case of ISOexpresso. Therefore, we anticipate that ISOexpresso will serve as a useful web-based platform for researchers who are not bioinformaticians to collect, examine, test, and hypothesize transcript-level changes in the tumorigenesis of various cancers.

## Construction and content

### ISOexpresso construction and implementation of functions

We constructed a web-based platform, ISOexpresso, which implements two main functions, Isoform Expression View and User Data Annotation, by integrating publicly available data and related information from various databases (See below).

#### Data acquisition and preparation

RNA-seq data (level 3, RNA-seq v2 expression data) and clinical information were obtained from The Cancer Genome Atlas (TCGA) Data Portal [19] for total 10,234 samples (9,499 tumors and 735 normal tissues) of 30 cancer types (see Additional file 1: Table S1). Tumor samples annotated as “primary solid tumor” and “recurrent solid tumor,” and matched normal samples as “solid tissue normal” were included in the 10,234 samples, whereas “metastatic” sample type was excluded. The estimated fraction of transcripts made up by a given isoform or gene ranging from zero to one computed using RSEM [5] was used to determine the statistical parameters (lower outliers, minimum, the first quartile (Q1), median, the third quartile (Q3), maximum, and upper outliers) for the expression levels of each gene and its isoforms in each sample group. If two or more expression levels of gene or isoform were found for the same patient, an averaged value was used in calculation of the above parameters. Median transcripts per million (TPM) was calculated by multiplying the median value of the estimates in each sample group by one million, which was then used to determine the major isoforms in the given tissue and sample types. If median TPM values of all isoforms of a gene were zero, Q3 values were compared between them to determine the major isoform.

#### Gene and isoform information

ISOexpresso basically follows gene and isoform definitions from UCSC Annotation database. At first, corresponding gene and isoform lists based on the hg19/GRCh37 reference genome were downloaded from the database (refGene.txt.gz, knownCanonical.txt.gz, knownGeneTxPep.txt.gz, and knownGene.txt.gz [20]). UniProt Retrieve/ID mapping page (idmapping.dat.gz [21]) was used for cross-referencing gene and isoform IDs among different annotation databases that provide their own specialized information, which included UniProt release 2015_08, RefSeq release 71, Ensembl release 74, and CCDS release 19. HUGO Gene Nom enclature Committee (HGNC) was used to enrich gene function annotation and to support the search module (i.e. searching with gene symbol, RefSeq ID, UniProt ID, Ensemble gene ID, and UCSC isoform ID [22]).

For most known genes, one to several isoforms have been regarded as representative of the normal state. Two main definitions are used in ISOexpresso. One is the “principal” isoform defined by the annotating principal splice isoforms (APPRIS) web database, which has been constructed by integrating protein structural information, functionally important residues, and evidence from cross-species alignments [23]. We downloaded the hg19/GRCh37 version (appris_data.principal.txt [24]) to annotate the corresponding isoforms. The other is the “canonical” isoform defined by the UniProt and UCSC Annotation databases. The UniProt-based canonical isoform is determined by considering all the protein products encoded by a gene for an isoform that satisfies at least one of the following criteria: i) it is the most prevalent; ii) it is most similar to orthologous sequences found in other species; iii) by virtue of its length or amino acid composition, it allows the clearest description of domains, isoforms, polymorphisms, and post-translational modifications. If no relevant information is available, the longest sequence is selected [25]. The UCSC-based canonical isoform can be identified in the knownCanonical.txt.gz file for each gene, which is generally the longest isoform [26].

#### Inference of tumor-specific isoforms

We implemented an inference module to help users identify tumor-specific isoforms that are only present or significantly over-expressed in tumors. It should be noted that since there are no consensus criteria on the inference methodology, this module is intended for supplemental use only. We considered two types of candidate tumor-specific isoforms. Type I includes the isoforms that are expressed in tumor samples but absent in the matching normal samples. To determine the expression status (expressed or unexpressed), we used a minimum threshold of median TPM >10^−6^, as used by Barrett et al. [10]. Type I candidates are further subdivided into two classes, strong and weak, where the median TPM value and the expression percentage of a strong candidate are greater than or equal to 1.0 and 10 %, respectively. Type II candidates are isoforms that are identified to be major in the tumor but not in the matching normal sample with a sufficient (median TPM fold change > 2) change in expression level.

#### Database construction, web deployment, and user interface

ISOexpresso uses MySQL (version 5) to store preprocessed expression levels of genes and isoforms as well as information gathered from public databases and web pages. It was developed using PHP language (version 5) and deployed onto web using Apache HTTP server (version 2.0). JavaScript and Highcharts [27] written in pure JavaScript were used for convenient user interface in ISOexpresso.

### Data analysis and applications

#### Consistency between major and canonical/principal isoforms

Among the 30 TCGA cancer types, 16 types for which 10 or more normal samples each were available were used for analysis. We selected one major isoform for each of the 26,541 genes based on the calculated isoform expression. Preselected canonical and principal isoforms (See Methods, Gene and isoform information) were compared to the major isoform for a consistency check. For each cancer type, the numbers of “matches” (the major isoform is the canonical/principal isoform) and “mismatches” were calculated using a custom Perl script (the script is available upon request). It should be noted that only the genes having at least one isoform with median TPM value greater than zero were included in the consistency check.

#### Variability of mutation consequence annotation

We applied the ISOexpresso User Data Annotation function to annotate major isoform for 144,160 somatic variants (VCF) called from 376 TCGA stomach adenocarcinoma (STAD) samples. Based on the annotation, we examined the possible discrepancies in predicting the consequence (alteration at the protein level) of each mutation when different transcript backbones were considered: expression-independent canonical/principal isoform vs. the major isoform. Using the downloaded VCF file, we first identified the genes at the chromosomal positions with sequence variations, and then determined the major isoform for each gene. Next, we checked the presence of mismatch between the major isoform and the available canonical/principal isoforms. For mismatched cases, we identified the position of each variant at the structural level of the transcripts, including exon (E), intron (I), 5^′^ or 3^′^ untranslated region (5U or 3U), or outside of transcript region (OTR). If a variant was located in an exon or intron, the number indicating the order of exons or introns from the transcription start site was also used to represent the variant location. We grouped the cases by comparing variant location in the major isoform and canonical/principal isoforms. For the five most frequently observed cases, we further investigated whether variant annotation at the protein level was changed when the major isoform was used instead of the canonical isoform.

## Utility

We implemented ISOexpresso with two main functions. First, ISOexpresso retrieves gene isoforms and their expression profiles for a given condition (Isoform Expression View function). In this function, users can query a gene of interest to find its known isoforms with annotations, check the most expressed isoform (the major isoform), compare expression profiles among different sample groups, and finally determine the condition-specific isoforms. Second, ISOexpresso automatically annotates a given mutation list (VCF) with matching isoform expression data to help users select a specific transcript for predicting the functional effects of the mutations (User Data Annotation function). By employing ISOexpresso, we further analyzed over 10,000 TCGA samples to showcase its practical use in inspecting tissue- and disease-specific isoforms, and the effects of transcript variability in mutation interpretation.

### Overall workflow of ISOexpresso

**Fig. 1:**
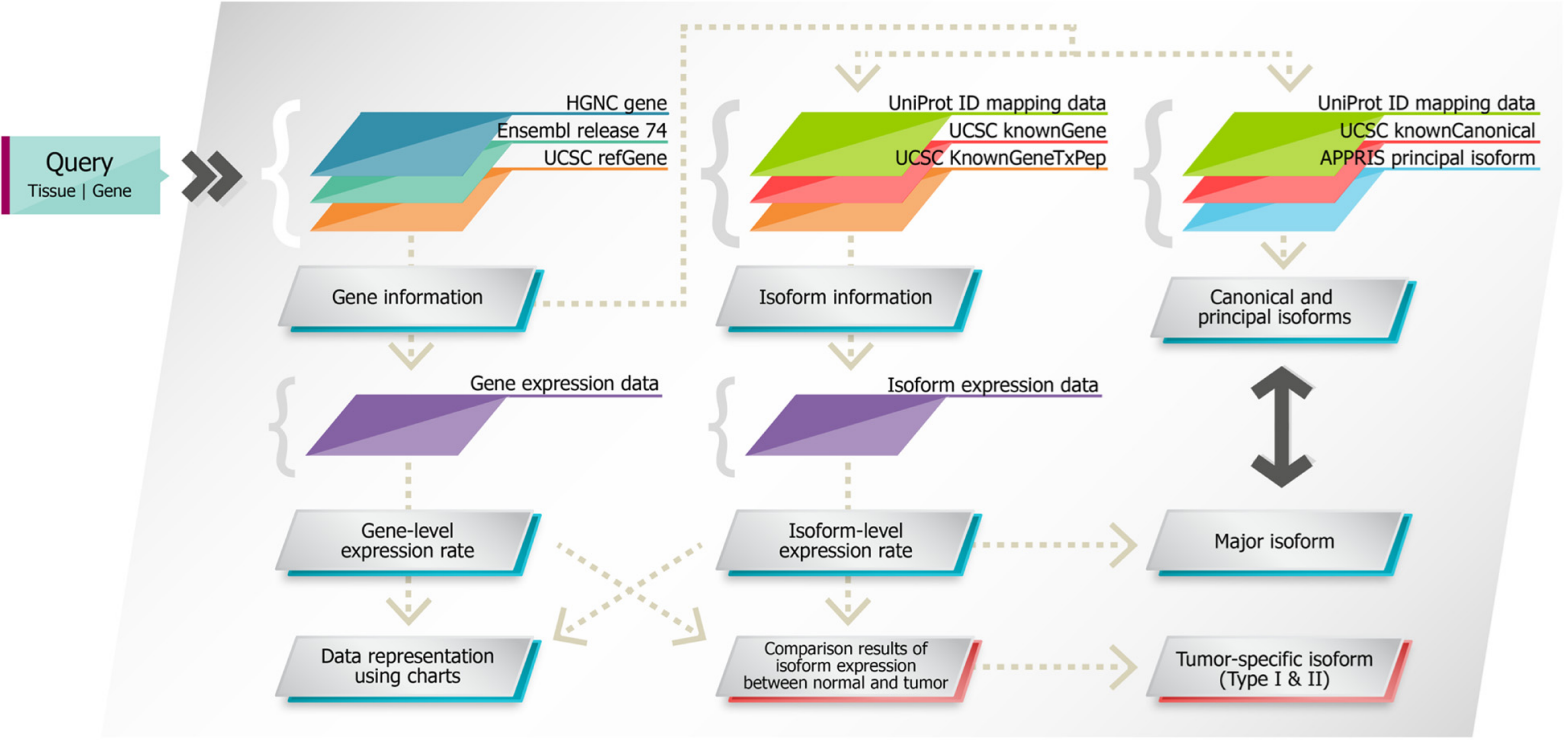
Overall workflow of ISOexpresso. Gene information is first determined by using user-provided query (tissue and gene). Isoform information, gene- and isoform-level RNA expression data, canonical and principal isoforms, and the major isoform are then obtained by searching corresponding database tables. Two functions of ISOexpresso (Isoform Expression View and User Data Annotation) are operated by this workflow

The overall workflow from user query to data output and visualization is shown in Fig. 1. When given a gene name with tissue information, ISOexpresso attempts to find a matched gene from three nomenclature tables (HGNC gene, Ensembl release 74, and UCSC refGene). Known isoform information of the gene is then collected from UniProt ID mapping data and UCSC knownGene. To determine whether the retrieved isoforms are protein-coding, ISOexpresso checks the availability of amino acid sequence information in the UCSC (knownGeneTxPep) table.

RNA expression levels of the matched gene and isoforms are extracted from prebuilt internal tables (constructed during the data acquisition step), which contain pre-calculated statistical data for each group (normal or tumor). These values are then processed for visualization using a box plot and stacked column chart. ISOexpresso selects the major isoform that is most expressed under a given condition (individual sample or disease/tissue group), and presents it along with the known canonical and principal isoforms for comparison. Finally, candidate tumor-specific isoforms are inferred based on the expression patterns.

### Visualization of isoform expression

**Fig. 2:**
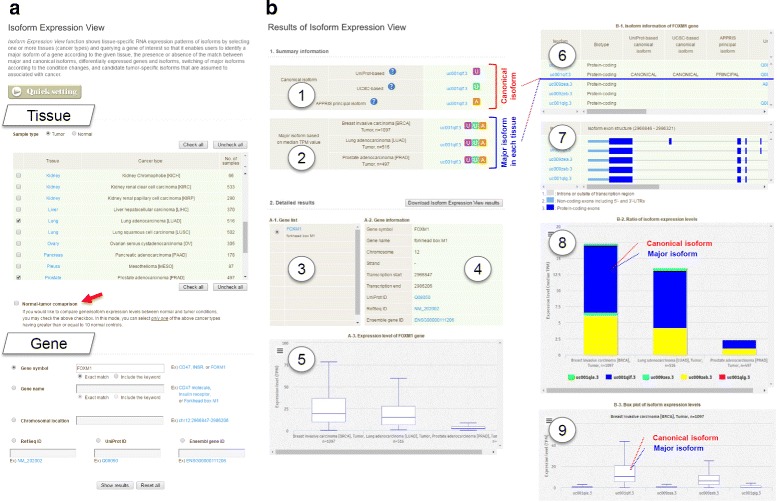
An example of using the Isoform Expression View function. **a** Start page, in which users can select one or more tissues and provide a gene to be searched. **b** Result page is consisted of i) summary information and ii) detailed results. Summary information shows canonical and principal isoforms of the gene (no. 1) and major isoforms for the selected tissues (cancer types; no. 2), in which two “U” squares in purple and green background mean that corresponding major isoforms are matched to UniProt-based and UCSC canonical isoforms, respectively, and a “A” square in orange background represent that the major isoform is identical with APPRIS principal isoform. Detailed results include gene list (no. 3), gene information (no. 4), gene expression level represented by box plot (no. 5), isoform information (no. 6), exon structure of each isoform (no. 7), ratio of isoform expression levels represented by stacked column chart (no. 8), and isoform expression levels represented by box plot (no. 9)

**Fig. 3:**
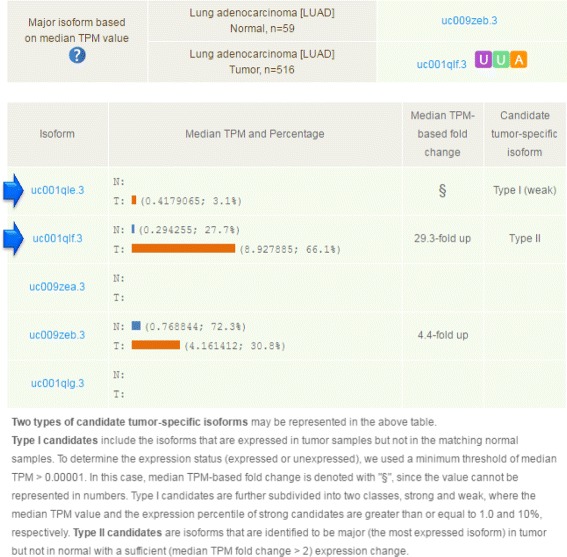
An example of inferring candidate tumor-specific isoform. This figure shows the comparison of isoform expression levels of FOXM1 between normal and tumor tissues in the LUAD cancer type. Two types of candidate tumor-specific isoforms are observed: the first isoform, uc001qle.3, is identified as a Type I (weak) candidate, and the second isoform, uc001qlf.3, is detected as a Type II candidate due to the switch of major isoform under tumor condition with a median-based fold change of 29.3-fold upregulation

**Fig. 4:**
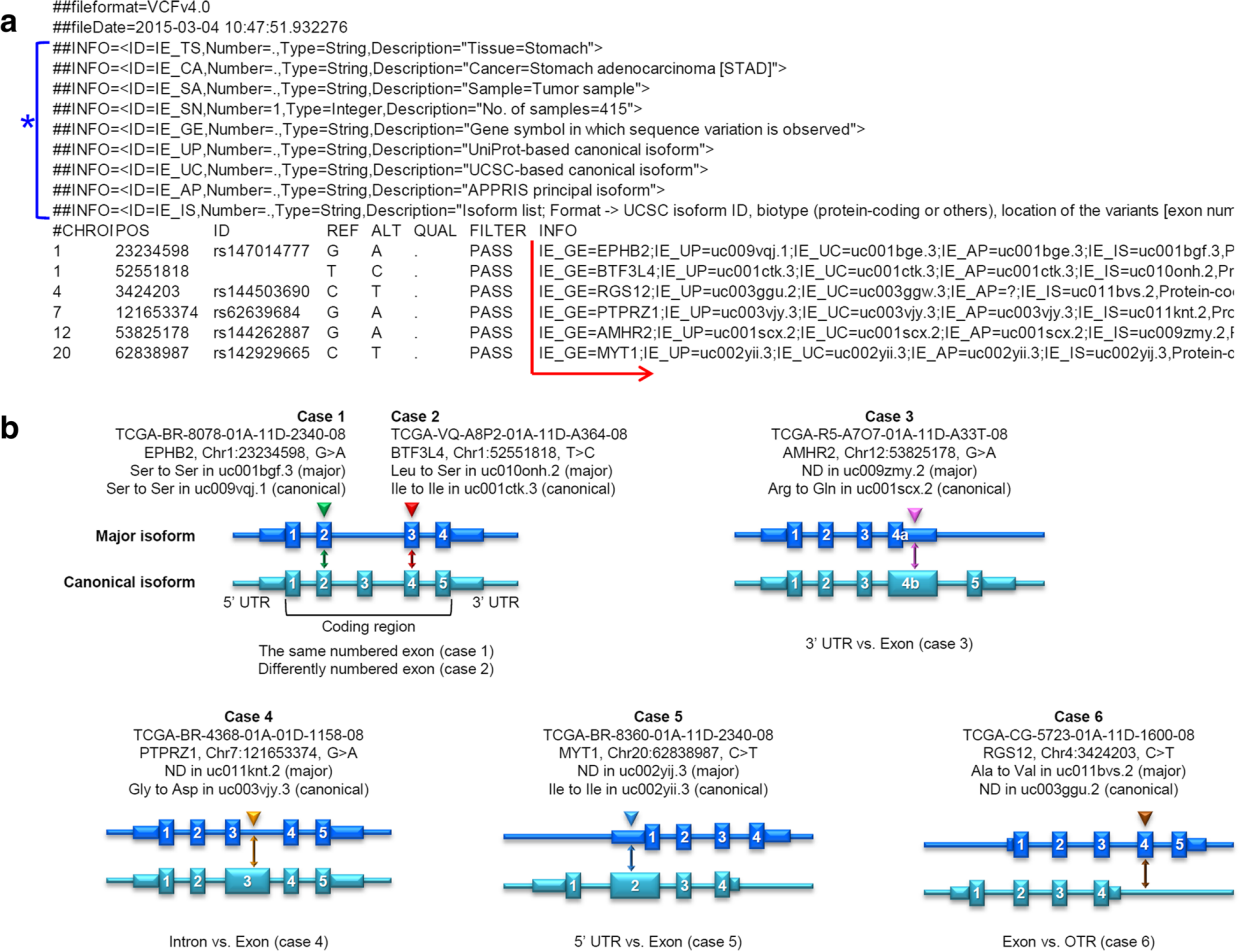
Example of a result page produced by the User Data Annotation function. **a** Output VCF file generated by the function, in which 9 information fields are newly added as indicated by the blue asterisk. **b** Most frequently observed cases when compared mutation consequences between major and canonical/principal isoforms using somatic mutations of TCGA STAD data. For each case, the TCGA sample barcode, gene symbol, chromosomal location, base change, and respective amino acid changes in the major and canonical isoforms are shown

**Fig. 5:**
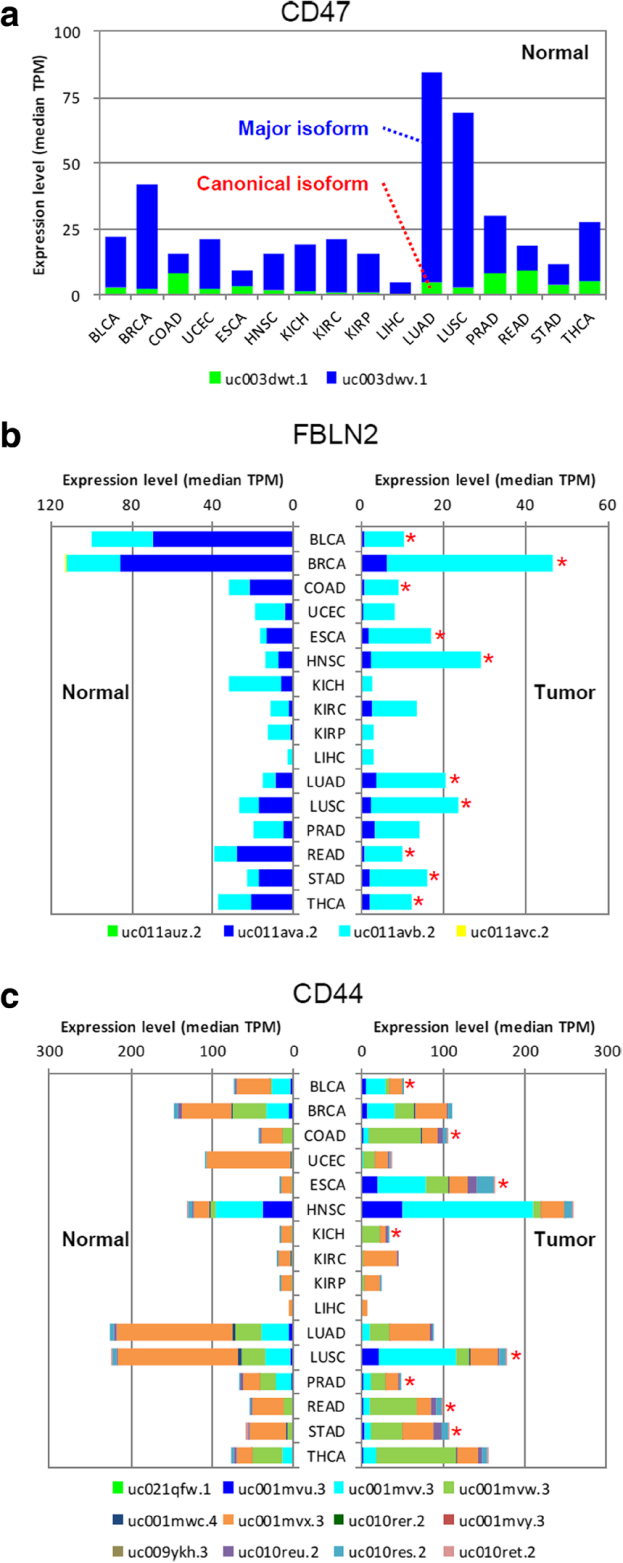
Isoform expression patterns that have been reported by previous studies. **a** The non-canonical major isoform (uc003dwv.1, blue) of CD47 is predominantly expressed in most of normal tissues compared to the canonical isoform (uc003dwt.1, lime). **b** FBLN2 (fibulin 2) exhibits switching of the major isoform (uc011ava.2, blue and uc011avb.2, cyan) between normal and tumor tissues. **c** Differential expression patterns of CD44 isoforms is shown across tissues, where one of the isoforms (uc001mvv.3, uc001mvw.3, or uc001mvx.3) is highly expressed as a major type in each tissue. In addition, major isoform switching during tumorigenesis was also found as represented by red asterisks

The overall summary of the Isoform Expression View is illustrated in Figs. 2 and 3. To start a new session, users have to submit their queries consisting of two types of information: tissue(s) and a gene of interest (Fig. 2a). Gene information may be provided in one of the following formats: gene symbol (e.g., FOXM1), gene name (e.g., Forkhead box M1), chromosomal location (e.g., chr12:2966847-2986206), RefSeq ID (e.g., NM_202002), UniProt ID (e.g., Q08050), or Ensembl gene ID (e.g., ENSG00000111206). Both keyword search and exact match modes are available. Based on the user query, the Isoform Expression View function outputs summary information and detailed results in a separate panel (Fig. 2b). Summary information shows the canonical isoforms (UniProt-based and UCSC-based) and the APPRIS principal isoform (Fig. 2b-1) together with the most expressed isoform (the major isoform) in the selected tissues (Fig. 2b-2). Here, users can quickly compare whether there is expression of any unexpected isoform. Detailed results display the selected gene list (Fig. 2b-3) and basic gene information (Fig. 2b-4). Expression at the gene level is compared between the selected tumor groups and visualized by a box plot (Fig. 2b-5). Next, a list of isoforms and their annotations are shown in a table to provide biotype (e.g. protein-coding), canonical and/or principal information, matching UniProt ID, RefSeq ID, Ensembl transcript ID, CCDS ID, and genomic coordinate (e.g. chr12:2966846-2986321), number of exons, and the length of each transcript (Fig. 2b-6). Exon structures of the isoforms are then visualized in a separate table (Fig. 2b-7), depicting protein-coding regions, 5^′^ and 3^′^ untranslated regions (UTRs), and introns. To visualize isoform-level expression and to compare the expression pattern across the selected tumors, ISOexpresso provides two additional graphic panels (Fig. 2b-8 and 9). First, the absolute expression levels (TPM) of all isoforms are presented in stacked column charts grouped by the selected tumor types. In the example provided in Fig. 2b-8, five known isoforms of the FOXM1 gene (uc001qle.3, uc001qlf.3, uc009zea.3, uc009zeb.3, and uc001qlg.3) are expressed in three cancer types (BRCA, LUAD, and PRAD). Based on the chart, one can easily determine that the known canonical isoform, uc001qlf.3, is the major isoform in all three cancer types, while a non-canonical isoform, uc009zeb.3 is also expressed at high levels. Furthermore, two additional isoforms are expressed at low levels in two cancer types (uc001qle.3 in BRCA and LUAD, and uc009zea.3 in BRCA). Following the grouped analysis, additional box plots are shown (Fig. 2b-9, only BRCA is shown here as an example) for each cancer type to visualize isoform-level expression. Users can easily check statistical parameters such as min, max, Q1, Q3, and median for further comparison within a cancer type. In addition, all the exact values are automatically presented in a hover box upon a mouseover event, and active URL link is supported for all database IDs (UCSC, UniProt, RefSeq, Ensembl, and CCDS) of the corresponding gene and its isoforms for users’ convenience. Furthermore, users can download Isoform Expression View results as tab-delimited text file by clicking a button of “Download Isoform Expression View results” that is located in the top-right side of detailed results (upper side of Fig. 2b-4), which information may be useful for integrating into pipelines or combining with results from other programs.

### Finding tumor-specific isoforms

ISOexpresso provides a simple module for searching isoforms that are conditionally expressed in cancer or normal samples (see Methods for detailed procedure). To enable this module, users have to select only one cancer type with ≥ 10 matched normal samples, and to check the “Normal-tumor comparison” mode in the query page (Fig. 2a, red arrow). Given an appropriate query, ISOexpresso shows one additional table in the standard visualization page (immediately below the summary information) that contains the result of the tumor -specific isoform analysis. An example is shown in Fig. 3. The table mainly shows a list of isoforms of the gene of interest, expression levels of each isoform in normal and tumor samples (in TPM), TPM-based fold changes, and provisional decisions on cancer specificity. We currently use two distinct criteria to annotate cancer specificity: one is the cancer-specific expression (only expressed in cancer, absent in normal, marked as Type I), and the other is the switch of the major isoform (most expressed in cancer but not in normal tissue, marked as Type II; see Methods for details). In the given example, uc001qle.3 is a Type I candidate, as it is expressed at approximately 0.40 TPM in the tumor and is absent in the normal samples. It is further classified as the “weak” type due to its low expression level in the tumor (< 1.0 TPM and (< 10 % of overall expression in the tumor). Likewise, uc001qlf.3 is a Type II candidate, because it is the major isoform only in cancer (66.4 %), while another isoform (uc009zeb.3) is the major isoform in the normal samples (77.6 %). As there is no consensus on the exact definition of tumor -specific isoform, we would like to note that additional information such as sample number, fold change, and gene function should be considered for users’ final decision. Nevertheless, ISOexpresso provides useful information for generating an initial hypothesis for the designated purpose.

### Mutation annotation for isoform-specific interpretation

Sequence variations in DNA usually affect phenotypes through changing amino acid sequences of proteins. In most cases, single nucleotide variant is interpreted into the corresponding amino acid change in a straightforward manner using a representative (canonical) transcript sequence. However, the consequence of variant can be dramatically changed if isoforms of different structures are considered, particularly when non-canonical isoforms are the major ones in a sample. We noted that the consequence of each DNA mutation must be interpreted with respect to the isoform-level expression profile for more accurate analysis. ISOexpresso provides the User Data Annotation function for this purpose. The User Data Annotation function can be run by simply selecting a tissue type with a user-uploaded variant list (VCF). When given a query, ISOexpresso outputs a newly annotated VCF with a downloadable link.

An example of results produced by this function is shown in Fig. 4a. In the output VCF file, nine novel fields are newly created (Fig. 4a, blue asterisk). These include 1) sample tissue (IE_TS), 2) cancer type considered for isoform expression (IE_CA), 3) sample type (IE_SA, tumor or normal), 4) the number of samples (IE_SN), 5) gene symbol at the mutation site (IE_GE), 6) UniProt-based canonical isoform (IE_UP), 7) UCSC-based canonical isoform (IE_UC), 8) APPIRS-based principal isoform (IE_AP), and 9) an isoform list sorted by expression level in the corresponding cancer type (IE_IS). Among them, the first four fields (IE_TS, IE_CA, IE_SA, and IE_SN) are directly annotated in the VCF header area, and the others (IE_GE, IE_UP, IE_UC, IE_AP, and IE_IS) are written in the entry of each variant (Fig. 4a, red arrow). For each isoform in the sorted isoform list (IE_IS), further information is included such as biotype (e.g. protein-coding), genetic elements at the position (e.g. E2: exon 2, I7: intron 7, 5U: 5^′^-UTR, 3U: 3^′^-UTR, and OTR: outside of transcription region), median TPM value of the isoform, and cross-reference database IDs including UniProt, RefSeq, Ensembl, and CCDS. With the sorted list, users can select the isoform that will be used for variant annotation (see Additional file 2: Figure S1).

We present several representative cases from TCGA data, in which isoform expression information is considered for variant annotation (Fig. 4b). In case 1, amino acid change is not altered (Ser to Ser), despite that the major isoform is not canonical. In case 2, the coding region of the T >C variant of BTF3L4 is different (exon 3 vs. 4) when considering the major isoform (uc010onh.2) and the canonical isoform (uc001ctk.3). While the former results in an amino acid change from Leu to Ser, the latter is interpreted as a synonymous mutation. In case 3, the G >A mutation in AMHR2 is interpreted as an Arg to Gln alteration in the canonical isoform (uc001scx.2), but it lies in the 3^′^-UTR region when the major isoform (uc009zmy.2) is considered. Likewise, cases 4 to 6 show that mutations can be interpreted in different ways, such as synonymous or non-synonymous mutation vs. non-coding mutations (intron, UTR, or OTR), depending on the isoform structure of the major transcript. We will show that a great number of variants are interpretable in different ways and they fall into one of the six cases (see Application 3).

### Application 1: Correspondence between the major and the canonical isoforms

As a case study, we inspected the overall consistency between the major isoforms and the canonical/principal isoforms in cancer (Table 1 and Additional file 3: Table S2). We examined the isoform expression levels of 26,541 genes from 15 cancer types under both normal and tumor conditions. For each cancer type, only expressed genes (median TPM > 0) were used for analysis (17,532–19,526 genes, 66.1–73.6 % of 26,541). In approximately 12 % of the expressed genes (1,867–2,547), no canonical or principal isoforms were defined. In the remaining genes (expression level is > 0 and canonical/principal isoform is defined), we tested whether the canonical/principal isoforms were the most expressed. Surprisingly, we found that the canonical/principal isoform matched the major isoform in only approximately 70 % (67.7–76.3 %) of the genes. In other words, non-canonical isoforms were more highly expressed in approximately 30 % of the genes (23.7–32.2 %).

**Table 1:** Statistics for the match and mismatch between major and canonical (or principal) isoforms

	No. of genes (*n*=26541)					
Cancer types	No. of samples	mTPM >0				Percent increase ^*b*^
		mTPM=0				
			Matched ^*a*^	Not matched		
				kCanon	nokCanon	
KIRP_normal	32	7359	12721 (2438) ^*c*^	3972 (1113)	2489 (1594)	
KIRP_tumor	290	8089	11544 (2474)	4580 (1266)	2328 (1475)	15.3 %
COAD_normal	41	7751	12562 (2543)	3919 (1189)	2309 (1597)	
COAD_tumor	460	8234	11805 (2442)	4291 (1298)	2211 (1443)	9.5 %
THCA_normal	59	7647	12389 (2363)	4019 (1071)	2486 (1489)	
THCA_tumor	505	7964	11838 (2405)	4387 (1166)	2352 (1504)	9.2 %
PRAD_normal	52	7429	12546 (2493)	4104 (1077)	2462 (1565)	
PRAD_tumor	497	7645	12042 (2549)	4445 (1228)	2409 (1523)	8.3 %
READ_normal	10	7402	12743 (2451)	3954 (1152)	2442 (1686)	
READ_tumor	165	8140	11879 (2499)	4283 (1277)	2239 (1459)	8.3 %
UCEC_normal	35	7715	11641 (2284)	4763 (1191)	2422 (1518)	
UCEC_tumor	545	7746	11226 (2515)	5152 (1323)	2417 (1551)	8.2 %
BLCA_normal	19	7986	11820 (2373)	4433 (1206)	2302 (1577)	
BLCA_tumor	408	8057	11381 (2541)	4791 (1412)	2312 (1593)	8.1 %
KIRC_normal	72	7411	12712 (2423)	3941 (1098)	2477 (1604)	
KIRC_tumor	533	7623	12166 (2387)	4240 (1174)	2512 (1627)	7.6 %
LUSC_normal	51	7251	12681 (2415)	4055 (1058)	2554 (1702)	
LUSC_tumor	502	7350	12404 (2563)	4300 (1187)	2487 (1670)	6 %
STAD_normal	35	7866	12901 (2559)	3443 (1151)	2331 (1663)	
STAD_tumor	415	7374	13017 (2520)	3608 (1196)	2542 (1723)	4.8 %
HNSC_normal	44	7831	12622 (3040)	3844 (1225)	2244 (1678)	
HNSC_tumor	520	7881	12383 (2788)	3994 (1193)	2283 (1626)	3.9 %
LUAD_normal	59	7802	12228 (2185)	4146 (1069)	2365 (1533)	
LUAD_tumor	516	7547	12263 (2401)	4274 (1163)	2457 (1622)	3.1 %
KICH_normal	25	7166	12787 (2420)	4020 (1098)	2568 (1599)	
KICH_tumor	66	8362	11776 (2773)	4146 (1368)	2257 (1529)	3.1 %
LIHC_normal	50	9136	10714 (3129)	4786 (1963)	1905 (1504)	
LIHC_tumor	370	8787	10782 (2860)	4907 (1769)	2065 (1534)	2.5 %
BRCA_normal	114	7396	12504 (2193)	4107 (1025)	2534 (1559)	
BRCA_tumor	1097	7711	12224 (2378)	4190 (1162)	2416 (1575)	2 %
ESCA_normal	11	7544	12824 (2847)	3701 (1306)	2472 (1772)	
ESCA_tumor	184	7136	13037 (2639)	3754 (1201)	2614 (1739)	1.4 %

^*a*^The number of genes whose major isoform is matched to at least one of UniProt-based and UCSC-based canonical and APPRIS principal isoforms

^*b*^Percent increase in the number of genes corresponding to “kCanon” when condition is changed from normal to tumor

^*c*^The numbers in parentheses indicate the number of genes whose expression level (mTPM) is greater than or equal to 1

mTPM = median transcripts per million

kCanon = the genes whose canonical or principal isoform is known

nokCanon = the genes whose canonical or principal isoform is not known

We postulate two reasons for the unexpectedly high expression of non-canonical isoforms. First, the expression levels or prevalence of isoforms is only part of the factors that define canonical and principal isoforms. In many cases, sequence conservation, length, expression or description clarity of domains or posttranslational modifications are also considered [28]. For these genes, the canonical or principal isoforms are not necessarily the most highly expressed. Second, there may be an actual change in isoform expression pattern under the specific condition. We found that the number of cases with non-canonical major isoforms was higher in the tumor than in the normal samples (Table 1 and Additional file 3: Table S2). In all 15 cancer types, the increase was consistently observed, ranging from 1.4 to 11.5 % (64 to 568 genes). Such result indicates that there may be aberrant expression of isoforms in cancer.

### Application 2: Rediscovery of unusual isoform expression patterns

In the majority of cases, the canonical isoforms are expected to be predominantly expressed across different tissues and conditions. However, there are also many unusual cases in which isoform expression does not follow the expected pattern. We tested whether ISOexpresso can showcase such unusual patterns reported by previous studies. Three genes that are known to show distinct isoform expression patterns were selected for ISOexpresso analysis. First, CD47 is a transmembrane protein that participates in a wide range of cellular process including apoptosis, proliferation, adhesion, and migration [29]. CD47 has been reported as a gene for which non-canonical isoform (ENST00000361309; uc003dwt.1) is predominantly and recurrently expressed in 16 tissues (adipose, adrenal, brain, breast, colon, heart, kidney, liver, lung, lymph node, ovary, prostate, skeletal muscle, testes, thyroid, and white blood cells) [30]. We could quickly confirm such pattern in 15 different tissues using the ISOexpresso Expression View function, five of which have not been reported previously (bladder, uterus, esophagus, head/neck, and stomach, Fig. 5a). The major (uc003dwv.1) and canonical (uc003dwt.1) isoforms were also readily identified. Second, we inspected the *FBLN2* gene that encodes an extracellular matrix protein with tumor-suppressive function [31]. A switch of major isoform during tumorigenesis has been reported [32], which was clearly demonstrated in ISOexpresso (Fig. 5b). Among the four known isoforms, two isoforms showed dramatic expression shift: from uc011ava.2 to uc011avb.2 in nine cancer types (Fig. 5b, red asterisks). For the third example, CD44 is a cell surface glycoprotein that plays crucial roles in development as well as in cancer progression and metastasis [33, 34], whose isoforms are known to be differentially expressed across tissues in many variant forms [35–37]. As expected, the expression pattern was reproduced in ISOexpresso (Fig. 5c). Thirteen of 15 normal tissues revealed that major type was an isoform, uc001mvx.3 (left chart of Fig. 5c). By contrast, one of three isoforms (uc001mvv.3, uc001mvw.3, or uc001mvx.3) was expressed as major type in tumor tissues (right chart of Fig. 5c). In addition, *CD44* also showed that major isoform switching was found in 7 tumor tissues during tumorigenesis represented as asterisks in right chart of Fig. 5b, showing heterogeneous expression of CD44 isoforms by aberrant RNA splicing in tumor.

### Application 3: Variability in mutation interpretation

We determined the extent to which DNA variation is subject to altered interpretation when different isoform structures were considered. We analyzed 147,540 somatic mutations called from TCGA STAD data with the ISOexpresso User Data Annotation function. By using UCSC-based canonical isoforms, we found 27,655 mutations for which the major isoform was not canonical (see Additional file 4: Figure S2). Among them, 25,544 (92.4 % of 27,655) were located in coding regions with respect to the canonical isoform structures.

We further inspected the possible alteration in interpretation of the 25,544 mutations by changing the backbone structure to the major isoform. We found that 21,599 (84.6 % of 25,544) mutations were still located in coding regions. Among the remaining 3,945 (15.4 %) mutations, 1,308 (5.1 %), 492 (1.9 %), and 2,145 (8.4 %) were located in intronic, 5^′^ UTR, and 3^′^ UTR regions of the major isoform, respectively. Therefore, consequences of these mutations were drastically changed when the major isoforms were considered for interpretation. Among the 21,599 mutations that were located in both the canonical and major isoforms, 9,373 (43.4 % of 21,599) were not accompanied by a change in exon number (as shown in Fig. 4b, case 1). Consistent with this, the amino acid residue for each mutation was predicted to be the same as that in the major isoform. In the other 12,226 mutations, exon number was changed (as shown in Fig. 4b, case 2). Among them, only 743 (0.6 % of 12,226) mutations that caused alteration in amino acid residue were interpreted to different amino acid; even the exon number has been changed, amino acid sequence is unaffected in translation if the reading frame was retained. We performed similar analyses using different definitions of canonical or principal isoform (UniProt and APPRIS), and produced similar results (see Additional file 4: Figures S3, S4).

## Discussion

ISOexpresso is designed to facilitate expression-based isoform-level analysis in cancer. To achieve this, RNA-seq data sets of 30 cancer types were collected from TCGA, which is a publicly available large-scale data source. ISOexpresso implements two main functions:, Isoform Expression View and User Data Annotation. The first function focuses on delivering condition- or tissue-specific isoform expression patterns (Fig. 2b), which enables detection of the major isoform in a given tissue, inspection of match or mismatch between the major and canonical isoforms, and provides visualization of differentially expressed genes and isoforms across distinct conditions or tissues. This function allows us to observe three different isoform expression patterns as described earlier (Fig. 5): i) recurrently observed major isoform across the tissues, ii) differentially expressed isoforms across the tissues, and iii) major isoform switching between normal and tumor conditions. Some of the isoforms corresponding to the second or third case may reflect characteristics of certain states of samples and can have potential as biomarkers for diagnosis [4, 10], cancer subtyping [11, 12], and tumor vaccine targets for immunotherapeutics [10]. For this purpose, we included a simple inference module in ISOexpresso for determining tumor-specific isoforms based on relative isoform expression between normal and tumor conditions, as shown in Fig. 3. The second function was implemented to offer annotation results of user-provided variants via expression-based prioritization of gene isoforms, thereby helping users to predict the effects of the variants. Figure 4b shows that isoform expression can be attributed as an influencing factor on variant effect prediction, where variant annotations in cases 2–6 were changed when using major isoforms instead of the canonical ones. In particular, case 2 reveals that a single variant can produce different results depending on which isoform is used as the reference for variant annotation: nonsynonymous missense mutation (leucine to serine) in the major isoform vs. synonymous mutation (isoleucine to isoleucine) in the canonical isoform. The above examples clearly demonstrate the usefulness and application of ISOexpresso in expression-based isoform-level analysis.

ISOexpresso allowed us to show the overall landscape for whether the major isoform of each gene matched the known canonical (or principal) isoform in normal and tumor tissues. Although the occurrence was low and the numbers varied depending onto the cancer types, we could observe a trend that the number of genes expressed as non-canonical major isoforms is increased by tumorigenesis as shown in Additional file 3: Table S2. This is clear evidence demonstrating the presence of differentially expressed isoforms and major isoform switching between normal and tumor tissues. As reported by previous studies [2, 3], cancer is closely associated with aberrant regulation of alternative splicing and therefore to the production of non-canonical and tumor-specific isoforms. Among the switched major isoforms in tumors, if their median TPM-based fold changes were greater than or equal to 2-fold compared to the normal samples, the isoforms were considered as Type II candidate tumor-specific isoforms, such as the second isoform in Fig. 3. Therefore, the overall survey revealed that ISOexpresso represents a useful tool to help discover candidate tumor-specific isoforms in various cancer types.

Several variant annotation tools are currently available, such as VEP [38], ANNOVAR [39], SeatleSeq [40], and snpEff [41]. The programs provide users with mainly mutation-oriented information such as nucleotide and amino acid changes corresponding to the sequence variations and mutation types (e.g., missense, nonsense). Although all transcripts are considered for variant annotation, there is no criterion to prioritize certain transcripts for predicting variant consequences. The User Data Annotation function of ISOexpresso prioritizes isoforms based on their relative expression levels, thereby helping users select an appropriate isoform for effective variant annotation. In addition, it provides not only the biotype of each isoform that enables the user to distinguish whether the particular isoform is protein-coding, but also known isoform IDs from several databases that make it possible to utilize previously annotated results using other tools. This additional information also helps the user select the most plausible isoform with functional consequence. Furthermore, it is possible to assist clinicians in making decisions by identifying the variant that is critical for patient treatment.

Regarding Isoform-level expression analysis, we searched currently available programs using RNA-seq data as following: Alt Event Finder [42], ARH-seq [43], ASprofile [44], DiffSplice [45], FDM [46], MATS [47], SplicingCompass [48], and SpliceSeq [49] for identifying transcripts that produced by alternative splicing in a gene or expressed as differential spliced forms between samples or conditions; RSEM [5], eXpress [6], Cufflinks [7], StringTie [8], and Sailfish [9] for estimating their expression level at transcript level; and Cuffdiff [7], EBSeq [50], rSeqDiff [51], and Ballgown [52] for extracting differentially regulated transcripts according to the condition change. For the purpose, it may be needed appropriate selection and combinational use of the programs. Among them, SpliceSeq has similar functions with ISOexpresso’s ones, which enables to support tabular and graphical visualization of transcripts and comparison between sample groups using SpliceSeq Viewer, and also uses a database system for storing and visualizing RNA-seq data of which the analysis is already completed. Moreover, the program allows users to analyze raw sequence data by using SpliceSeq Analyzer. Accordingly, SpliceSeq may also be appropriate for isoform-level RNA-seq data analysis. Nevertheless, we expect that ISOexpresso may be more specified for cancer genome research compared to SpliceSeq, since it allows users to provide isoform expression data for multiple cancer types based on large-scale cancer database (TCGA). In addition, any users can access ISOexpresso to extract information regarding isoform existence and expression, since it allows anonymous access on the web.

TCGA is a project aimed to study the DNA and RNA of multiple cancer types, and it releases publicly available high throughput sequencing data. TCGA data have been incorporated into recent studies. However, several cancer types have few or no matched normal tissue samples. The comparison between normal and tumor samples is restricted if only TCGA data sets are used. A project named the Genotype-Tissue Expression (GTEx) aims to provide the scientific community with a resource to study human gene expression and regulation, and their relationships with genetic variations [53]. The GTEx also releases all project data through the database of Genotypes and Phenotypes (dbGaP [54]), and summary data on the GTEx portal. These expression data from normal tissues will be incorporated into our ISOexpresso database in the near future. Therefore, we expect that it will be possible to reliably present candidate tumor-specific isoforms upon obtaining sufficient amounts of expression data in normal tissues.

Data update is a crucial issue for maintaining a database. TCGA data sets have updated and released on TCGA Data Portal. Accordingly, update of data sets included in ISOexpresso database should be performed for maintenance of newly released data. We have a plan of regular data update quarterly. This work also contains gradual conversion of gene and isoform expression data based on new version of reference genome (Hg38/GRCh38) instead of Hg19/GRCh37 version. Moreover, uploading and processing of user’s own expression data generated from RSEM will be supported in the near future, which may be a useful ISOexpresso function for researcher. Therefore, isoform-level analysis in ISOexpresso will be achieved by providing newly updated TCGA data and by supporting analysis from user’s own data.

## Conclusion

ISOexpresso was designed to facilitate expression-based isoform-level analysis of large-scale TCGA multi-cancer RNA-seq data. There are two main functions in ISOexpresso: i) the Isoform Expression View function focusing on delivering condition- and tissue-specific isoform expression levels and ii) the User Data Annotation function prioritizing variants based on their relative expression levels to help predict the most plausible functional variant. We also confirmed that the ISOexpresso functions could reproduce the results reported by previous studies. Therefore, ISOexpresso will be a useful web-based platform for isoform-level analysis in cancer.

## Availability and requirements

**Database:** ISOexpresso **Database homepage:**http://wiki.tgilab.org/ISOexpresso/**Operating system(s):** Microsoft Windows, Linux, or Mac OS **Programming language:** PHP and JavaScript **Other requirements:** Apache, PHP, and MySQL

These data are freely available without restrictions for use by academics.

## Abbreviations

3U, 3’-UTR; 5U, 5’-UTR; ACC, adrenocortical carcinoma; APPRIS, annotating principal splice isoforms; BLCA, bladder urothelial carcinoma; BRCA, breast invasive carcinoma; CCDS, the Consensus Coding Sequence project; CESC, cervical squamous cell carcinoma and endocervical adenocarcinoma; CHOL, cholangiocarcinoma; COAD, colon adenocarcinoma; dbGaP, the database of genotypes and phenotypes; FBLN2, Fibulin 2; FOXM1, Forkhead Box M1; ESCA, esophageal carcinoma; GBM, glioblastoma multiforme; GTEx, the Genotype-Tissue Expression; HGNC, HUGO Gene Nomenclature Committee; HNSC, head and neck squamous cell carcinoma; KICH, kidney chromophobe; KIRC, kidney renal clear cell carcinoma; KIRP, kidney renal papillary cell carcinoma; LAML, acute myeloid leukemia; LGG, brain lower grade glioma; LIHC, liver hepatocellular carcinoma; LUAD, lung adenocarcinoma; LUSC, lung squamous cell carcinoma; MESO, mesothelioma; OTR, outside of transcription region; OV, ovarian serous cystadenocarcinoma; PAAD, pancreatic adenocarcinoma; PCPG, pheochromocytoma and paraganglioma; PRAD, prostate adenocarcinoma; Q1, the first quartile; Q3, the third quartile; READ, rectum adenocarcinoma; RefSeq, the reference sequence; RNA-seq, transcriptome (RNA) sequencing; STAD, stomach adenocarcinoma; TCGA, The Cancer Genome Atlas; TGCT, testicular germ cell tumors; THCA, thyroid carcinoma; THYM, thymoma; TPM, transcripts per million; UCEC, uterine corpus endometrial carcinoma; UCS, uterine carcinosarcoma; UCSC, the University of California, Santa Cruz; UniProt, the Universal Protein Resource; UTR, untranslated region; UVM, uveal melanoma; VCF, variant call format

## Additional files

Additional file 1
**Table S1.** TCGA RNA-seq data of 30 cancer types. (XLSX 12.7 kb)

Additional file 2
**Figure S1.** Detailed explanation of the results produced by the User Data Annotation function for the T >C variant of BTF3L4 (case 2 in Fig. 4 b). (PNG 75.3 kb)

Additional file 3
**Table S2.** Overall landscape of the match and mismatch between the major and canonical (or principal) isoforms in the 15 cancer types. (PDF 697 kb)

Additional file 4
**Figure S2, S3, and S4.** Variability check results in mutation interpretation using TCGA STAD data. **Figure S2.** Cases in which the major isoform does not match the UCSC-based canonical isoform. **Figure S3.** Cases in which the major isoform does not match the UniProt-based canonical isoform. **Figure S4.** Cases in which the major isoform does not match the APPRIS principal isoform. (XLSX 25.2 kb)
