# Erratum to: ISOexpresso: a web-based platform for isoform-level expression analysis in human cancer

**DOI:** 10.1186/s12864-016-3180-6

**Published:** 2016-10-25

**Authors:** In Seok Yang, Hyeonju Son, Sora Kim, Sangwoo Kim

**Affiliations:** 1 Severance Biomedical Science Institute, Yonsei University College of Medicine, 50-1 Yonsei-ro, Seoul, 03722 Korea; 2 Brain Korea 21 PLUS Project for Medical Sciences, Yonsei University College of Medicine, 50-1 Yonsei-ro, Seoul, 03722 Korea

## Erratum

In the original publication of this article [1], the grant number HI15C1234 is incorrect and should be corrected to HI15C1601.

The correction details of the grant number can be found below:
