# Supplementary figures and images for: ISOexpresso: a web-based platform for isoform-level expression analysis in human cancer
Source: BMC Genomics. 2016 Aug 12;17:631. doi: 10.1186/s12864-016-2852-6 (PMC4983006; doi:10.1186/s12864-016-2852-6)

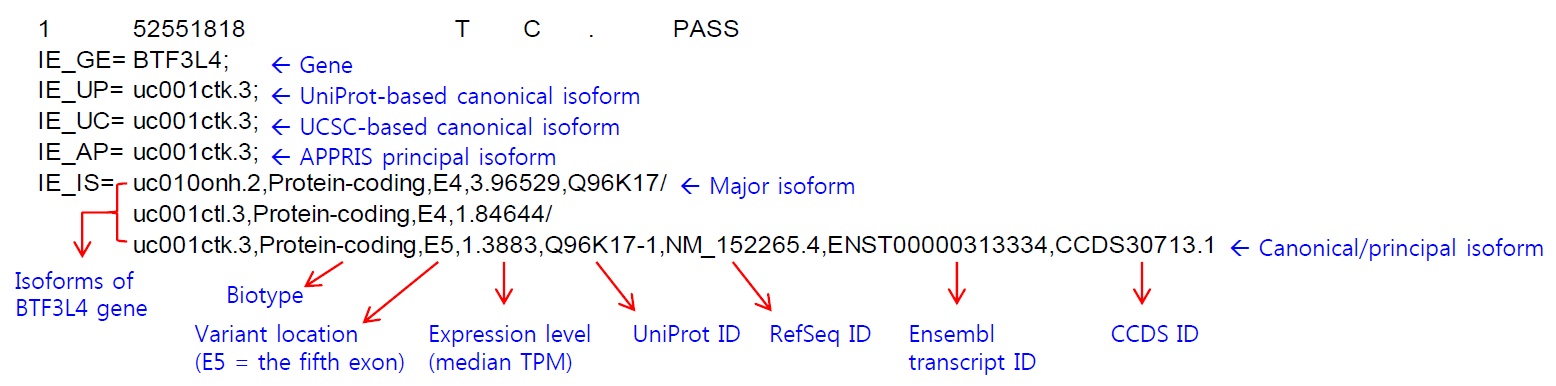

Supplement: Additional file 2 — Figure S1. Detailed explanation of the results produced by the User Data Annotation function for the T >C variant of BTF3L4 (case 2 in Fig. 4 b). (PNG 75.3 kb) [file 12864_2016_2852_MOESM2_ESM.png]
