# Supplementary material for: ISOexpresso: a web-based platform for isoform-level expression analysis in human cancer
Source: BMC Genomics. 2016 Aug 12;17:631. doi: 10.1186/s12864-016-2852-6 (PMC4983006; doi:10.1186/s12864-016-2852-6)
Supplement: Additional file 4 — Figure S2, S3, and S4. Variability check results in mutation interpretation using TCGA STAD data. Figure S2. Cases in which the major isoform does not match the UCSC-based canonical isoform. Figure S3. Cases in which the major isoform does not match the UniProt-based canonical isoform. Figure S4. Cases in which the major isoform does not match the APPRIS principal isoform. (XLSX 25.2 kb) [file 12864_2016_2852_MOESM4_ESM.pdf]

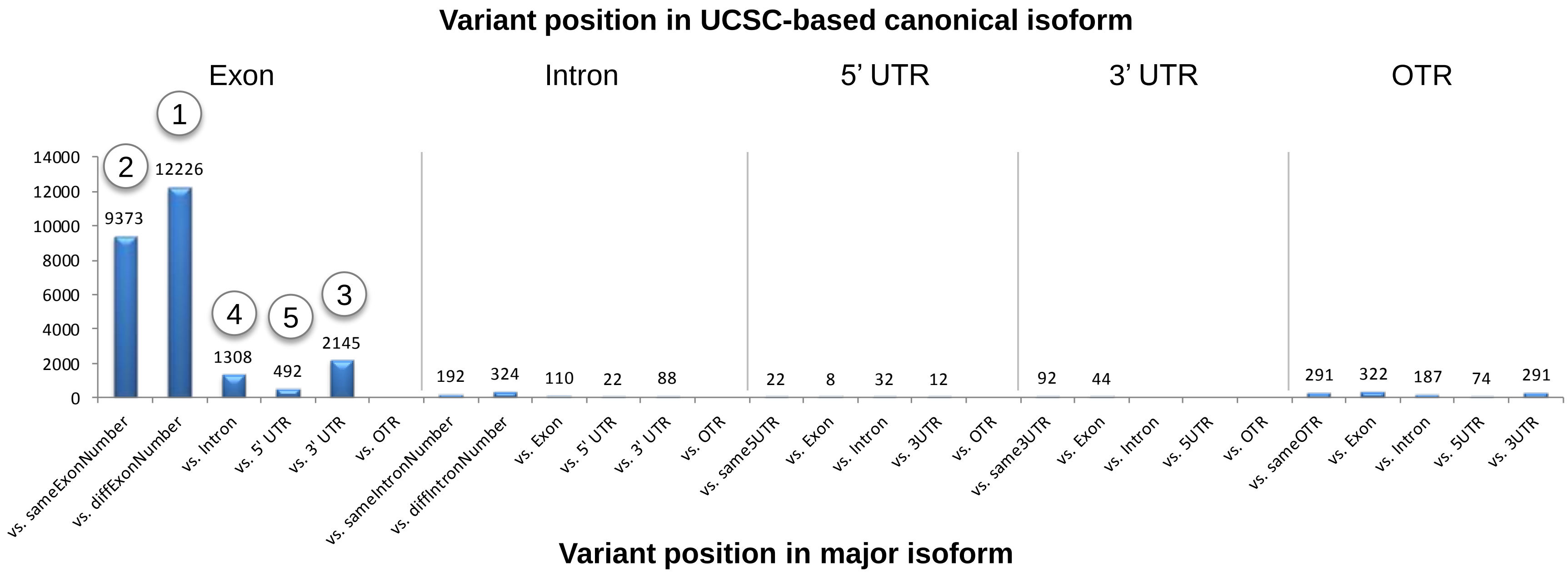


Total variants examined = 147,540
Total mismatched cases between major and canonical isoforms = 29,669
Mismatched cases that canonical isoform is available = 27,655

Rank 1 = 12,226 / 147,540 * 100 = 8.3% → Annotations were changed in 743 cases (0.5%).
Rank 2 = 9,373 / 147,540 * 100 = 6.4%
Rank 3 = 2,145 / 147,540 * 100 = 1.5%
Rank 4 = 1,308 / 147,540 * 100 = 0.9%
Rank 5 = 492 / 147,540 * 100 = 0.3%

Fig. S2

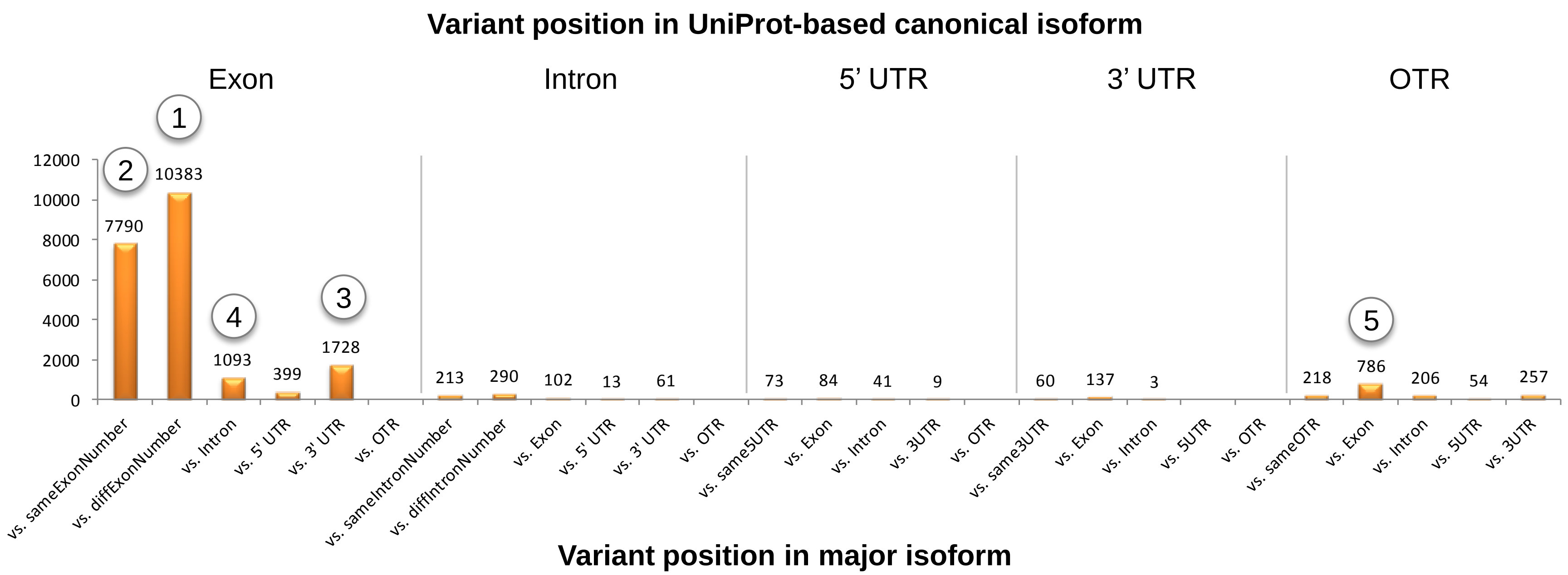


Total variants examined = 147,540
Total mismatched cases between major and canonical isoforms = 29,669
Mismatched cases that canonical isoform is available = 24,000

Rank 1 = 10,383 / 147,540 * 100 = 7.0% → Annotations were changed in 110 cases (0.07%).
Rank 2 = 7,790 / 147,540 * 100 = 5.3%
Rank 3 = 1,728 / 147,540 * 100 = 1.2%
Rank 4 = 1,093 / 147,540 * 100 = 0.7%
Rank 5 = 786 / 147,540 * 100 = 0.5%

Fig. S3

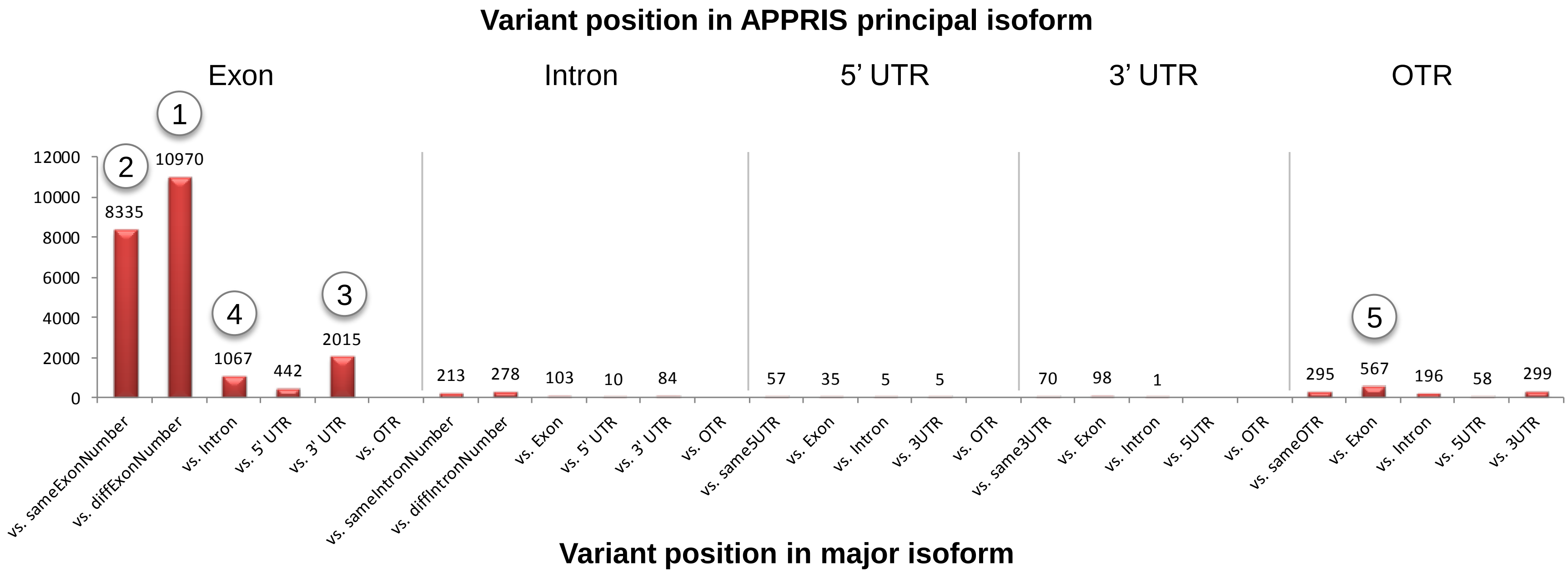


Total variants examined = 147,540
Total mismatched cases between major and principal isoforms = 29,669
Mismatched cases that canonical isoform is available = 25,203

Rank 1 = 10,970 / 147,540 * 100 = 7.4% → Annotations were changed in 467 cases (0.3%).
Rank 2 = 8,335 / 147,540 * 100 = 5.6%
Rank 3 = 2,015 / 147,540 * 100 = 1.4%
Rank 4 = 1,067 / 147,540 * 100 = 0.7%
Rank 5 = 567 / 147,540 * 100 = 0.4%

Fig. S4
